# The Potential Effect of Chinese Herbal Formula Hongqijiangzhi Fang in Improving NAFLD: Focusing on NLRP3 Inflammasome and Gut Microbiota

**DOI:** 10.1155/2018/5378961

**Published:** 2018-02-21

**Authors:** Shu Liang, Yupei Zhang, Yuanjun Deng, Yifang He, Yinji Liang, Zien Liang, Yanning Chen, Kairui Tang, Runsen Chen, Qinhe Yang

**Affiliations:** School of Traditional Chinese Medicine, Jinan University, Guangzhou 510632, China

## Abstract

The present study investigates the potential therapeutic mechanism underlying the effects of the Chinese herbal formula Hongqijiangzhi Fang (HJF) on nonalcoholic fatty liver disease (NAFLD) in rats. Male Sprague Dawley (SD) rats were randomly divided into 4 groups (*n* = 8): control group was fed a normal diet, three other groups were fed high-fat diets (HFD), and the two treatment groups were intragastrically given a compound probiotic or HJF during the molding time. After 16 w, related indices were detected. The results showed that HJF significantly reduced abdominal aorta serum cholesterol (TC), triglyceride (TG), low-density lipoprotein (LDL), IL-1*β*, and IL-18, portal venous serum lipopolysaccharide (LPS), and liver TC and TG levels in HFD-fed rats. HJF ameliorated hepatic steatosis in the liver and improved the intestinal barrier in HFD-fed rats. Activation of the NLRP3 inflammasome was reduced by HJF in HFD-fed rats. Additionally, the abundances of* A. muciniphila* (Verrucomicrobiaceae),* F. rappini* (Helicobacteraceae), and Enterobacteriaceae bacteria significantly decreased in HJF-treated HFD-fed rats. In conclusion, these result suggested that the Chinese herbal formula HJF reduced hepatic steatosis maybe through decreasing certain gut bacteria (such as Enterobacteriaceae bacteria and* F. rappini*), alleviating intestinal endotoxemia and reducing NLRP3 inflammasome activation.

## 1. Introduction

Nonalcoholic fatty liver disease (NAFLD) is characterized by hepatic steatosis, ruling out ethanol intake and other specific pathogenic factors [1]. The NAFLD prevalence rate is 25.24% worldwide, and this condition is closely related to diseases such as obesity, type 2 diabetes (T2D), hyperlipidemia, and metabolic syndrome; furthermore, NAFLD may develop into nonalcoholic steatohepatitis (NASH) and even liver cirrhosis and hepatocellular carcinoma (HCC) [2]. NAFLD also correlates with other systemic diseases, such as cardiovascular disease (CVD), chronic kidney disease (CKD), and inflammatory bowel disease (IBD) [3, 4].

The precise pathogenesis of NAFLD has still not been fully illustrated, although the most acceptable explanation is the “two hit” theory presented by Day and James in 1998 [5]. In recent years, the multiple parallel hits hypothesis had been proposed, which encompasses the impact of factors such as adipose tissue, genetic factors, inflammation, endotoxin, and the gut microbiota on NAFLD and suggests that these factors lead to hepatic steatosis, inflammation, and liver damage [6]. The transplantation of fecal microbiota samples from obese individuals into germ-free mice increases adiposity compared to the effects of transplantation from lean individuals [7]. Clinical research has shown that the gut microbiota is altered in NAFLD patients and changes with NAFLD severity [8]. Dysbiosis of the gut microbiota in NAFLD increases intestinal permeability, leading bacterial metabolites such as endotoxin (lipopolysaccharide [LPS]) to access to the liver [9, 10]. LPS activates the NOD-like receptor protein 3 (NLRP3) inflammasome in the liver, resulting in the production of proinflammatory factors such as IL-18/1*β* and accelerating the NAFLD process [11, 12]. The NLRP3 inflammasome consists of a cytoplasmic innate receptor (NLRP3), an apoptosis associated speck-like protein containing a CARD domain (ASC), and a cysteinyl aspartate specific proteinase (Caspase-1) [13]. It had been reported that activation of the NLRP3 inflammasome correlates with obesity and insulin resistance (IR) [14].

Traditional Chinese medicine (TCM) is an effective treatment for NAFLD [15]. Many herbal medicines prevent steatosis and NAFLD through various proposed mechanisms, and Chinese medicinal formulae combine multiple Chinese herbal medicines under the guidance of traditional Chinese medical theory [16]. Clinical and animal studies suggest that Chinese medicinal formulae decrease serum cholesterol (TC), triglyceride (TG), and low-density lipoprotein (LDL) in individuals with NAFLD [17, 18]. In a previous study, we showed that invigorating spleen recipes of Chinese medicine therapy reduce hepatic steatosis and inflammatory factors in rats fed a high-fat diet (HFD) [19]. Hongqijiangzhi Fang (HJF) is a Chinese medicinal formula that, according to invigorating spleen recipes, combines seven kinds of herbal medicine.

The aim of the present study was to determine the potential therapeutic mechanism of experimental treatment with HJF in HFD-fed rats. The HFD was administered for 16 w to induce NAFLD, as described in a previous study [20] with little modification. Then, we used various technologies and methods to detect changes in the gut microbiota, intestinal barrier integrity, portal serum LPS levels, NLRP3 inflammasome protein expression, and other related indices in HFD-fed rats with HJF intervention. The results demonstrated that the Chinese medicinal formula HJF exerted pleiotropic effects on NAFLD.

## 2. Materials and Methods

### 2.1. Animal Experiments

Six-week-old specific pathogen-free (SPF) Sprague Dawley (SD) male rats (weight: 200 ± 20 g, *n* = 32) were obtained from the Laboratory Animal Research Center of Guangzhou University of Traditional Chinese Medicine (license number SCXK (Yue) 2013-0034) and raised in an SPF animal center (12 h daylight cycle, temperature: 18°C–22°C) with free access to food and drink. The use of animals in this study was approved by the Ethics Committee of Medical College of Jinan University. After acclimation for 1 w, the rats were randomly divided into 4 groups (*n* = 8): the control group (NC group), HFD group, CP group, and CR group. The NC group was fed a normal diet, while the other three groups were fed a HFD (high-fat feed processing formula: 83% basic feed, 10% lard, 1.5% cholesterol, 0.5% bile salts, and 5% sucrose), which was processed by Guangdong Medical Laboratory Animal Center (license number SCXK (Yue) 2013-0002). The groups were simultaneously given pure water or medication by gastric gavage: the CP group received daily doses (0.6 g/kg) of a compound probiotic (Table 1) (obtained from Professor Heping Zhang, College of Life Science, Inner Mongolia Agricultural University, China), the CR group received daily doses (19.05 g/kg) of HJF (Table 2) (granules except Hongqu; Tianjiang Pharmaceutical Co, Ltd., China, batch number: 1603127) (Hongqu, used after grinding; Jun Tong Pharmaceutical Co, Ltd., batch number: 160302), and the NC and HFD groups received same volume of pure water. The treatment intervention period lasted 16 w. Body weight gain was assessed once a week. Fresh feces were collected one day before the animals were sacrificed following anesthesia. Abdominal aorta and portal venous blood, colon tissues, and liver tissues were collected for each rat. The livers were weighed, and the hepatic indices were counted.

### 2.2. Histology Determination (Oil Red O)

Frozen sections (thickness: 8 *μ*m) of fresh liver tissues were stained with Oil red O (Nanjing Mindit Biochemistry Co., Ltd., China) for 5 min and then counter-stained with hematoxylin for 3 min. Light microscopy was used to examine the sections. Image-Pro Plus 6.0 was used for positive rate measurement.

### 2.3. Biochemical Analyses and Cytokine Measurements

Abdominal aorta and portal venous blood were centrifuged at 3000 RPM for 10 min at 4°C, and the upper serum was collected. To obtain tissue homogenates, 100 mg of liver tissue was mixed with 0.9 mL of isopropanol, followed by centrifugation at 3000 RPM for 10 min at 4°C, and subsequently the tissue homogenate supernatant was collected. Abdominal aorta serum TC, TG, LDL, and high-density lipoprotein (HDL) levels, as well as liver TC and liver TG levels, were detected by using an automatic biochemical analyzer (Hitachi, Japan). Portal venous serum LPS levels were measured using a Quantitative Chromogenic Tachypleus Amebocyte Lysate (TAL) For Endotoxin Detection Kit (Chinese Horseshoe Crab Reagent Manufactory, CO., Ltd., China) according to the manufacturer's instructions. Abdominal aorta serum IL-1*β* and IL-18 levels were tested using enzyme-linked immunosorbent assay (ELISA) kits (the IL-1*β* ELISA kit was obtained from eBioscience, USA. The IL-18 ELISA kit was obtained from Invitrogen Corporation, USA).

### 2.4. Immunohistochemistry Assay

Colon tissues were fixed with formalin, followed by paraffin embedding and subsequent sectioning (thickness: 5 *μ*m) for an immunohistochemistry assay to examine the expression of occludin protein. Antibodies specific for occludin were obtained from USA Origene. Expression was determined using DAB detection kits (streptavidin-biotin) (ZSGB-BIO, China) according to the manufacturer's instructions.

### 2.5. Western Blotting

Western blotting was performed to detect the protein expression of NLRP3, ASC, and Caspase-1. Liver tissue homogenate was generated with a homogenizer after lysis and centrifugation at 14000 RPM for 10 min and 4°C. The supernatant was collected in a 1.5-mL EP tube. The protein content was determined with a BCA protein content test kit (KeyGEN BioTECH, China). The samples were separated by SDS-PAGE and transferred to a PVDF membrane, followed by washing with TBST and incubation with blocking buffer. After shaking for 60 min, anti-NLRP3 (Novus Biologicals, USA), anti-ASC (Novus Biologicals, USA), and anti-Caspase-1 (Abcam, UK) antibodies were added, and the membranes were incubated at 4°C for 16 h. After washing with TBST, a secondary antibody was added and incubated at 37°C for 1 h, followed again by washing. An HRP-labeled *β*-actin monoclonal antibody (1 : 10000, KangChen Bio-tech Inc., China) was used as an internal reference. After incubation with a secondary antibody, the membrane was placed in a clean cling film, followed by incubation with an appropriate amount of ECL chemiluminescent solution for 5 min. Absorbent paper was used to draw excess liquid from the membrane. The film was fixed in a film holder in a dark room for 1 min, followed by immediate development fixation. The exposure time was adjusted according to the time required for band imaging, and the imaging time was recorded.

### 2.6. 16s rDNA Genetic Sequencing and Bioinformatics Analysis

Genomic DNA extracted from feces samples was used to amplify the V3 and V4 regions of 16S rDNA by using specific primers (341F: CCTAYGGGRBGCASCAG, 806R: GGACTACNNGGGTATCTAAT). The PCR amplification products were gel-extracted and quantitated using a QuantiFluor™ fluorometer. A sequence connector was added, and a sequencing library was constructed after mixing equal amounts of the purified amplification products. A Hiseq2500 PE250 was used for computer sequencing.

The raw data were sequence-filtered to clean the data under two conditions: first, sequences with *N* base ratios greater than 10% in reads were removed; and second, reads with quality values above 20 bases and a total base percentage lower than 80% (base quality value *Q* = −10 · log 10(*e*), base quality value *Q* < 20 considered a base error rate larger than 1%) were also removed. Then, double-end reads were joined into a sequence based on the overlapping relationship between PE reads (minimum matching length: 10 bp, overlapping area mismatch rate allowed: 0.02), and the new sequence was called a tag. A Naive Bayesian method based on the rdp classifier tool was used to annotate tags with species. Mothur (v.1.34.0) was used to calculate the number of OTUs at a distance of 0.03 (97% similarity); additionally, the Shannon index was calculated, and Shannon rarefaction curves were drawn. Principal component analysis (PCA) and the heat map were performed by R software. Metastats software (using Fisher's exact test) was used to analyze the differences in relative abundance of gut microbiota between groups, and an inspection level of  FDR < 0.05  was considered statistically significant. The differences in the major flora between the groups were analyzed by using LEFse.

### 2.7. Statistical Analyses

SPSS for Windows 19.0 was used for data statistics, and GraphPad Prism 6.0 was used for drawing. The data are presented as the mean ± standard deviation (SD), and results were analyzed by one-way ANOVA between each group. An inspection level of *P* < 0.05 was considered statistically significant.

## 3. Results

### 3.1. HJF Demonstrates a Protective Effect in HFD-Fed Rats

**Figure 1:**
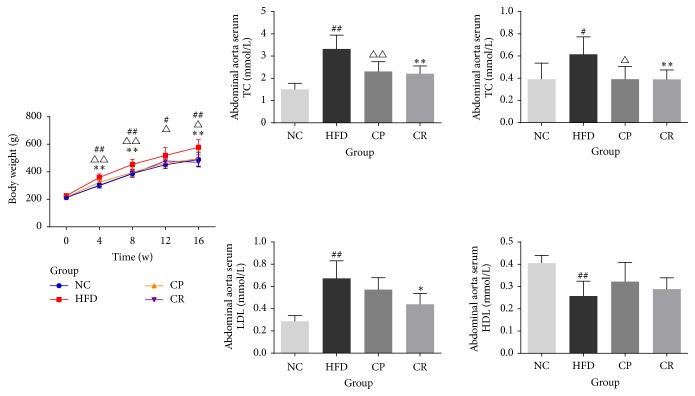
Weight curve, level of abdominal aorta serum TC, TG, LDL and HDL in NC, HFD, CP, and CR group. ^#^*P* < 0.05 NC group versus HFD group, ^##^*P* < 0.01 NC group versus HFD group, ^△^*P* < 0.05 CP group versus HFD group, ^△△^*P* < 0.01 CP group versus HFD group, ^*∗*^*P* < 0.05 CR group versus HFD group, and ^*∗∗*^*P* < 0.01 CR group versus HFD group, *n* = 8.

As shown in Figure 1, the weight curve for the HFD group was higher than those of the other three groups, except at 0 w. At 16 w, the weight of the rats in the HFD group was significant higher than that of the rats in the NC group (*P* < 0.01), and the weights of the rats in the CP and CR groups were obviously lower than those of the rats in the HFD group (*P* < 0.05 or *P* < 0.01). Abdominal aorta serum TC, TG, and LDL levels in the HFD group were significantly increased compared with those in the NC group (*P* < 0.05 or *P* < 0.01), and the CR group had lower levels of TC, TG, and LDL than the HFD group (*P* < 0.05 or *P* < 0.01). The CP group also had lower levels of TC and TG than the HFD group (*P* < 0.05 or *P* < 0.01), but LDL levels showed no obvious difference between these two groups (Figure 1). The HFD group had lower HDL levels than the NC group (*P* < 0.01), but the CP and CR groups exhibited no remarkable differences from the HFD group (Figure 1). These results indicated that HFD-fed rats demonstrated significantly increased body weight and disorder of lipid metabolism; CP and HJF ameliorated these effects in HFD-fed rats.

### 3.2. HJF Improves Hepatic Steatosis in HFD-Fed Rats

**Figure 2:**
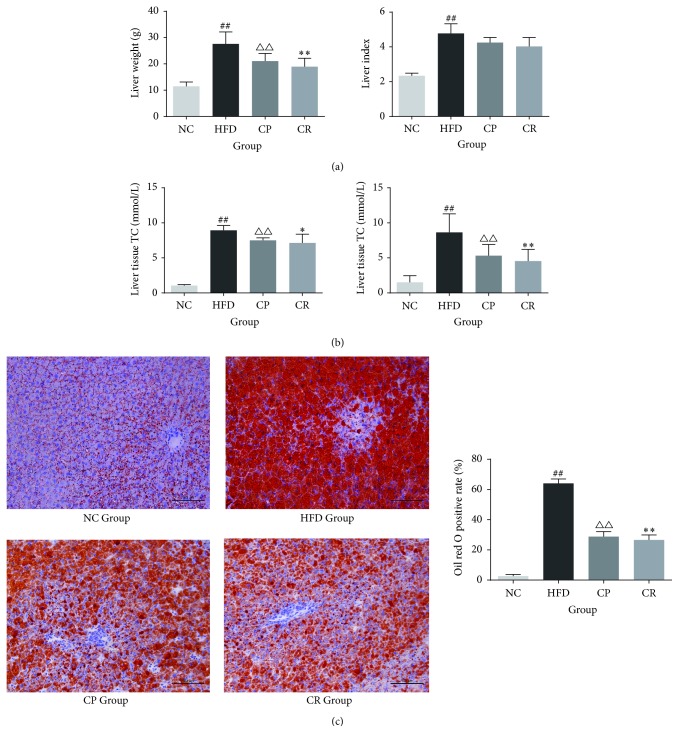
The hepatic steatosis of rats in NC, HFD, CP, and CR group: (a) Liver weight and liver index. (b) Liver tissue TC and TG. (c) Oil red O staining (stain ×200) and positive rate. ^##^*P* < 0.01 NC group versus HFD group, ^△△^*P* < 0.01 CP group versus HFD group, ^*∗*^*P* < 0.05 CR group versus HFD group, and ^*∗∗*^*P* < 0.01 CR group versus HFD group, *n* = 8.

As shown in Figures 2(a) and 2(b), the rats in the HFD group had heavier livers, larger liver indices, and higher liver tissue TC and TG levels than the rats in the NC group (*P* < 0.01), while the CP and CR groups demonstrated significant reductions in liver weight and liver TC and TG levels compared with those in the HFD group (*P* < 0.05 or *P* < 0.01). Oil red O staining and the positive rate of it (Figure 2(c)) showed that HFD group rats had serious hepatic steatosis, and this hepatic steatosis was notably alleviated in the CP and CR groups. Thus, HFD increases liver weight and causes liver hepatic steatosis in rats, and CP and HJF attenuate liver hepatic steatosis in HFD-fed rats.

### 3.3. HJF Alleviates HFD-Induced Endotoxemia

**Figure 3:**
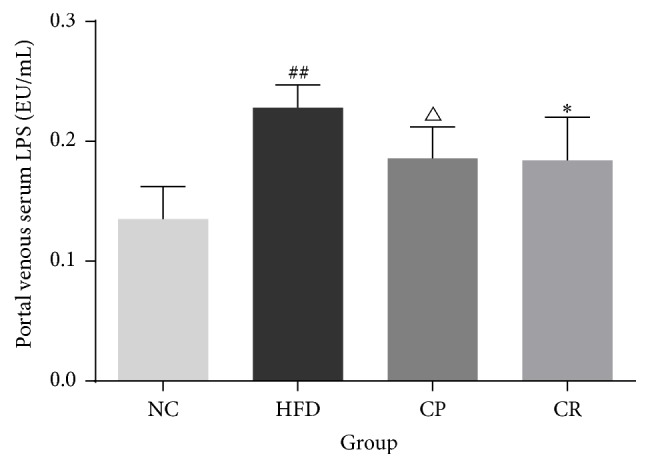
Level of portal vein serum LPS in NC, HFD, CP, and CR group. ^##^*P* < 0.01 NC group versus HFD group, ^△^*P* < 0.05 CP group versus HFD group, and ^*∗*^*P* < 0.05 CR group versus HFD group, *n* = 8.

Increased levels of portal vein serum LPS are a hallmark of endotoxemia; therefore, we measured the levels of portal vein serum LPS in the experimental rats (Figure 3). The HFD group had higher levels of LPS compared with the NC group (*P* < 0.01), and LPS levels in the CP and CR groups were decreased compared with that of the HFD group (*P* < 0.05). This result suggested that HFD-fed rats may have endotoxemia, and CP and HJF alleviate this condition to a certain extent.

### 3.4. HJF Improves Intestinal Barrier Integrity in HFD-Fed Rats

**Figure 4:**
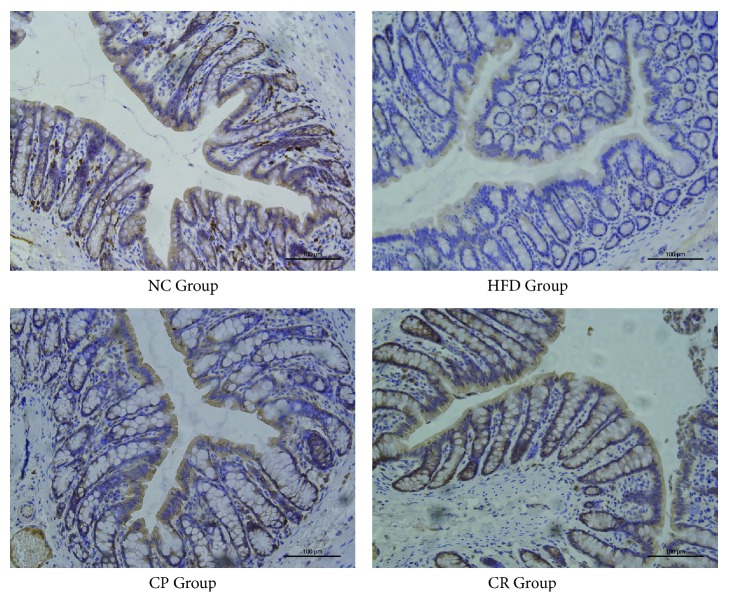
Immunohistochemistry staining for occludin in colon tissues of NC, HFD, CP, and CR group (stain ×200).

As a tight junction protein, occludin plays important roles in the intestinal barrier. A deficiency in these proteins may increase intestinal permeability. The immunohistochemistry results showed that occludin expression levels were reduced in colon tissues from HFD-fed rats, while the CP and CR groups exhibited different degrees of enhanced occludin expression levels (Figure 4). These results suggest that HFD increases intestinal permeability, and CP and HJF exerted positive effects on the intestinal barrier.

### 3.5. HJF Reduced NLRP3 Inflammasome Activation and Cytokine Release

**Figure 5:**
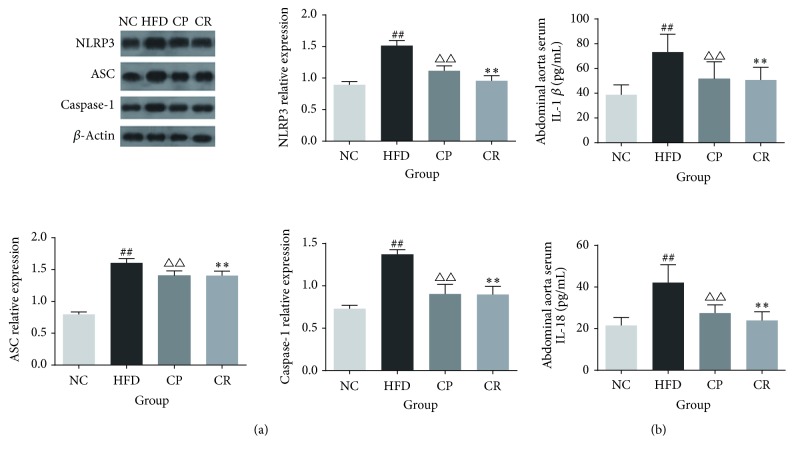
Expression of NLRP3 inflammasome related proteins and the level of related cytokinin in NC, HFD, CP, and CR group. (a) Expression of NLRP3, ASC, and Caspase-1 protein (*n* = 5). (b) Level of abdominal aorta serum IL-1*β* and IL-18 (*n* = 8). ^##^*P* < 0.01 NC group versus HFD group, ^△△^*P* < 0.01 CP group versus HFD group, and ^*∗∗*^*P* < 0.01 CR group versus HFD group.

The NLRP3 inflammasome consists of NLRP3, ASC, and Caspase-1. Western blotting (Figure 5(a)) showed that NLRP3, ASC, and Caspase-1 activation were significantly increased in the HFD group compared with the levels in the NC group (*P* < 0.01) and obviously decreased in the CP and CR groups (*P* < 0.01). Higher levels of serum IL-1*β* and IL-18 were observed in the HFD than in the NC group (*P* < 0.01), and the CP and CR groups had lower levels of serum IL-1*β* and IL-18 than the HFD group (*P* < 0.01) (Figure 5(b)). These results suggested that HFD up-regulates NLRP3 inflammasome activation and the release of IL-1*β* and IL-18, while HJF significantly reduces these effects.

### 3.6. HJF Alters the Gut Microbiota Structure in HFD-Fed Rats

**Figure 6:**
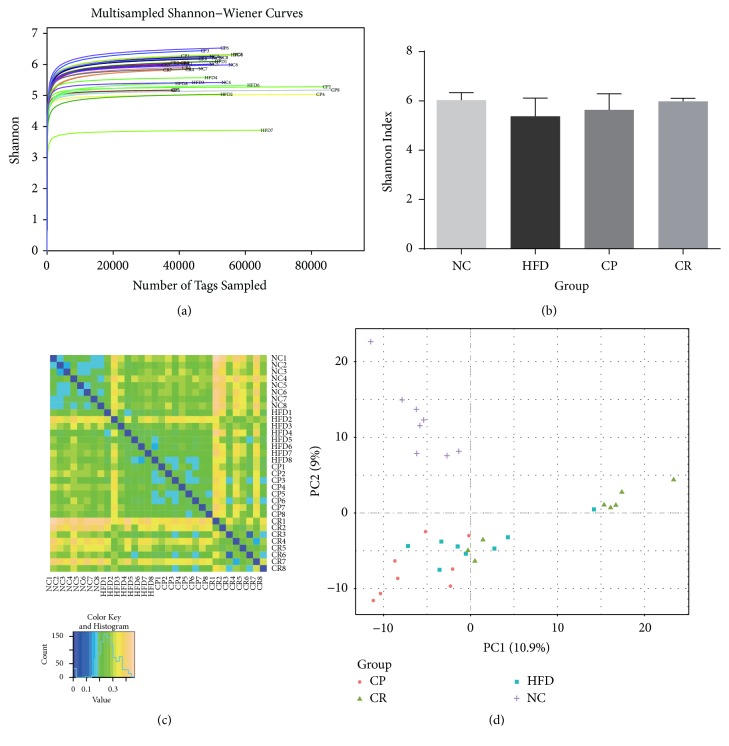
Abundance and diversity of gut microbiota in NC, HFD, CP, and CR group: (a) Shannon Curve. (b) Shannon Index. (c) Beta diversity. (d) PCA score plot.

As shown in Figure 6(a), the smooth Shannon rarefaction curves meant the sequencing depth was sufficient, and the majority of the gut microbes in each sample were detected. The Shannon index is a diversity index encompassing comprehensive OTU richness and OTU evenness, and the larger the Shannon index, the more abundant the species in the samples. The Shannon index in the CR group was larger than that in the HFD group, but the difference was not statistically significant (Figure 6(b)). Beta diversity reflects the difference in species diversity between different samples, as shown in Figure 6(c), and the difference between the CR and NC groups was highest, followed by the difference between the CR and HFD groups. The PCA results (Figure 6(d)) showed that the gut microbiota structure had changed, and most of the HFD group samples were concentrated in the third quadrant, while the remaining groups were concentrated in the first and the second quadrants, unlike the NC group samples, which were concentrated in the fourth quadrant. The CR group was primarily concentrated in the first quadrant, and the remaining groups were concentrated in the second and the third quadrants. According to the PCA results, the distribution of the CP group closely aligned with that of the HFD group (Figure 6(d)). These results suggest that although the abundance of bacterial species did not differ significantly between each group, there was a difference in species diversity, and the gut microbiota structure was altered by HJF in HFD-fed rats, although there were still differences from the gut microbiota structure in normal-diet-feed rats.

### 3.7. HJF Influenced Specific Gut Bacteria in HFD-Fed Rats

**Figure 7:**
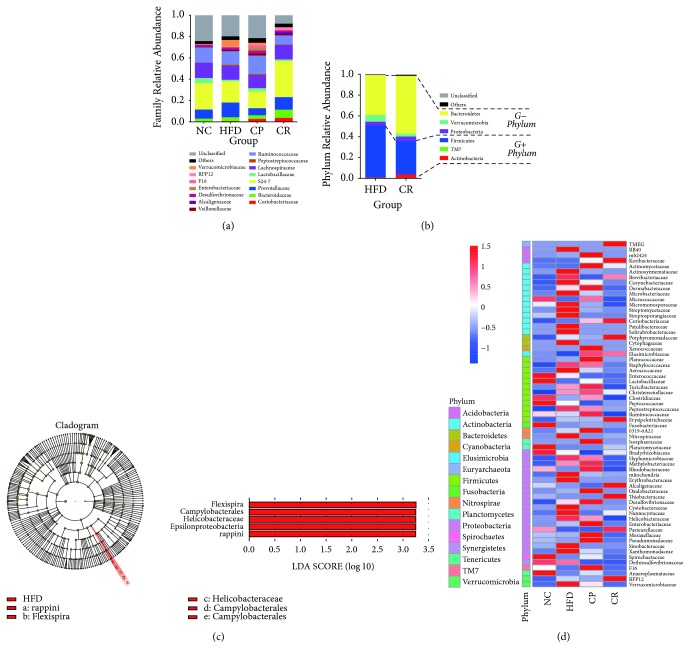
Alteration of gut microbiota in HFD-fed rats with intervention of HJF: (a) Bacteria Family Relative Abundance in four groups. (b) Gram-positive (G+) and Gram-negative (G−) bacterium Phylum Relative Abundance of HFD and CR group. (c) LEFse analysis of HFD group versus CR group. (d) Heat map of bacteria family with significant difference between each two groups (FDR < 0.05).

To determine the appropriate level for species classification, we counted the tag number sequences at each classification level. More than 58.42% of the tag sequences were annotated to the family level in each sample, but the annotated tag sequences at the genus level were lower than 15.95%. Thus, we selected the family level as the best classification level for the 32 analyzed samples because of the balance between the species level and the number of annotated tags. At the family level, the dominant bacterial families, such as Prevotellaceae,* S24-7*, Alcaligenaceae, and Ruminococcaceae, did not differ significantly between the NC, HFD, and CR groups (Figure 7(a)). We identified 16 bacterial families with obvious differences in the HFD and CR groups (FDR < 0.05) (Figure 7(d)). Among these, 15 families, including Helicobacteraceae (0.43% HFD versus 0.09% CR), Verrucomicrobiaceae (4.89% HFD versus 0.01% CR), and Enterobacteriaceae (0.37% HFD versus 0.07% CR), were decreased following HJF administration in HFD-fed rats. The difference in Helicobacteraceae between the HFD and CR groups primarily derived from* Flexispira rappini* (Helicobacteraceae) (0.43% HFD versus 0.09% CR). The difference in Verrucomicrobiaceae between the HFD and CR groups derived from* Akkermansia muciniphila* (Verrucomicrobiaceae) (4.89% HFD versus 0.01% CR).

LEFse analysis of the HFD group versus the CR group showed that “Bacteria-Proteobacteria-Epsilonproteobacteria-Campylobacterales-Helicobacteraceae-Flexispira-rappini” was the primary difference between these two groups, and HJF obviously reduced these bacterial numbers in HFD-fed rats (Figure 7(c)).

## 4. Discussion

In the present study, we administered a HFD to induce NAFLD in rats. Weight and abdominal aorta serum and liver tissue TC and TG levels were obviously increased in HFD-fed rats, suggesting a serious lipid metabolic disorder in these animals. Clinically, liver biopsy is the most reliable method to diagnose NAFLD [21]. Oil red O staining showed substantial liver fat deposits in HFD-fed rats, indicating that the NAFLD rat model was successfully established. We found that HJF, a TCM formula intended to strengthen the spleen, alleviated lipid metabolic disorders and reduced liver fat deposits in HFD-fed rats, suggesting that HJF exerted therapeutic effects on NAFLD. Since probiotic treatment is also effective for NAFLD [22], we used a compound probiotic as a positive control intervention in one group of HFD-fed rats in the present study. The results indicated that HJF had a similar effect as the compound probiotic in HFD-fed rats. Moreover, as demonstrated by the bacterial species diversity analysis, the gut microbiota structure was altered by HJF in HFD-fed rats, although it had not been restored to the kind of balance in rats fed a normal diet.

In present study, we detected two species* A. muciniphila* (Verrucomicrobiaceae) and* F. rappini (*Helicobacteraceae), and one family of bacteria, Enterobacteriaceae; those experienced significant decreases after intervention with HJF in HFD-fed rats. The abundance of* A. muciniphila* increased in HFD-fed rats in present study. In contrast, Schneeberger et al. reported that a HFD decreased the abundance of* A. muciniphila* in mice [23]. Zhao et al. reported that body weight and liver fat were decreased after treatment with* A. muciniphila* in mice fed a normal diet [24]. Although enrichment of the number of* A. muciniphila* was observed in T2D patients in one study [25], another clinical study showed that the abundance of* A. muciniphila* increased as the weight of T2D patients decreased after treatment with an antidiabetic [26]. This evidence indicates that* A. muciniphila* is a bacterial species that is beneficial for health. However, the influence of different diets on the gut microbiota has also been reported [27]. In the present study, the abundance of* A. muciniphila* was significantly increased in HFD-fed rats compared with rats in the NC group, and the HFD recipe used in the present study differed from that used in other studies; this might be a factor that contributes in part to the difference in* A. muciniphila* abundance. And no matter what, the treatment of HJF in HFD-fed rats is observed, but it might be independent of altering the abundance of* A. muciniphila*.

*Enterobacteriaceae*, a bacterial family within* Proteobacteria*, was previously shown to be increased in a NASH patient, consistent with high levels of serum LPS [28]. Singh et al. reported that this family of bacteria is associated with LPS production [29]. A major fermentation product of* Enterobacteriaceae* is alcohol [30]. Alcohol induces upper gastrointestinal injury and increased gut permeability [31]. An increase in endogenous ethanol and gut permeability was observed in NAFLD patients by Volynets et al. [32]. In the present study, although the abundance of Enterobacteriaceae did not significantly differ between the NC and HFD groups (*P* = 0.042, FDR = 0.168), HJF intervention obviously decreased the abundance of this bacterial family (*P* = 0.005, FDR = 0.049). Moreover, the intestinal permeability of the HFD-fed rats increased, showing that HJF treatment exerts a positive effect on intestinal barrier integrity. Thus, we speculate that HJF improves intestinal barrier integrity in part by reducing the abundance of Enterobacteriaceae in HFD-fed rats.

Furthermore, Enterobacteriaceae, which includes pathogenic bacteria such as* Salmonella*,* Shigella,* and* Escherichia coli*, comprises a minor proportion of the gut microbiota but has been associated with intestinal inflammation [33–35]. In vitro experiments have shown that* E. coli* strains that accumulate in the intestine of IBD patients induce the production of IL-1*β* via the NLRP3 inflammasome [36]. Another study showed that* Proteus mirabilis* (a member of Enterobacteriaceae) induces robust IL-1*β* via the NLRP3 inflammasome [37]. Increased gut permeability in HFD-fed rats was observed in the present study. Increased gut permeability leads to bacterial translocation, in which bacteria such as* E. coli* cross the mucosal barrier via the blood circulation to other organs, such as the liver and spleen [38–40]. Therefore, we hypothesized that Enterobacteriaceae bacterial translocation potentially activates the NLRP3 inflammasome and stimulates the release of IL-1*β* in the livers of HFD-fed rats. Additionally, HJF may reduce the translocation of Enterobacteriaceae to reduce activation of the NLRP3 inflammasome, thereby alleviating hepatic inflammation.

*F. rappini* (Helicobacteraceae) is also a member of Proteobacteria. However, until recently, there have been few studies investigating* F. rappini* and even fewer reports associating this bacterial species with NAFLD and related diseases. Sheng et al. reported that the abundance of Helicobacteraceae bacteria is increased in FXR knockout mice, accompanied by an increase in hepatic lipids and serum endotoxin [41]. Additionally, infection by* Helicobacter pylori*, a species of the Helicobacteraceae family, is associated with NAFLD [42]. He et al. showed that* H. pylori* infection aggravates central obesity and insulin resistance in HFD-fed mice [43]. The damaging effects of* H. pylori* on the gastrointestinal mucosal barrier have previously been reported [44]. But, we did not know whether* F. rappini* employs a similar mechanism as* H. pylori* during the development of NAFLD and the relationship between reduced* F. rappini* abundance and HJF treatment in HFD-fed rats. However, additional research is needed.

Clinical research has revealed increased levels of LPS in NAFLD patients [45]. Animal experiments have also demonstrated high levels of LPS in HFD-fed rats [46]. In the present study, similar results were observed in HFD-fed rats. LPS not only is a factor of inflammation but is also associated with obesity [47]. Cani et al. showed that the induction of LPS in mice fed a normal diet leads to lipid metabolism similar to that observed in HFD-fed mice [48]. Fukunishi et al. reported that the intraperitoneal injection of LPS exacerbated hepatic steatosis in Zucker (fa/fa) rats [49]. LPS is a major component of the cell walls of Gram-negative bacteria; however, in the present study, HJF decreased LPS levels, but this was not accompanied by a decrease in the abundance of Gram-negative bacteria in NAFLD rats (Figure 7(b)). Mao et al. described intestinal mucosal barrier dysfunction in NAFLD rats, which facilitated LPS access to the liver, induced hepatic immune cell activation, and exacerbated liver injury [50]. Given our observation of the positive effects of HJF treatment on intestinal barrier integrity in HFD-fed rats, we speculate that HJF lowers LPS levels; however, this occurs not through a decrease in the total abundance of Gram-negative bacteria but through improved intestinal barrier integrity.

LPS is involved in NLRP3 inflammasome activation through two mechanisms: (1) via recognition by TLRs, leading to NF-*κ*B pathway activation as one of two priming signals required for NLRP3 inflammasome activation and the subsequent up-regulation of NLRP3, pro-IL-1*β*, and pro-IL-18 expression, or (2) via binding to Caspase-11 in the cytosol via a noncanonical pathway, both mechanisms eventually lead to the activation of inflammation and induce the release of IL-1*β* and IL-18 [51–53]. In the present study, we observed that the liver NLRP3 inflammasome is activated, and serum IL-1*β* and IL-18 levels are increased in HFD-fed rats. Consistent with these findings, activation of the NLRP3 inflammasome has been correlated with HFD-induced hepatic steatosis [54]. As previously mentioned, the NLRP3 inflammasome consists of NLRP3, ASC, and Caspase-1. Stienstra et al. reported that NLRP3/ASC/Caspase-1 deficiency reduced HFD-induced obesity in mice, IR and hepatic steatosis were alleviated in ASC-deficient HFD-fed mice, and the absence of Caspase-1 similarly alleviated IR [55]. Dixon et al. reported that HFD-fed Caspase-1 knockout mice had lower levels of hepatic steatosis and inflammation [56]. Activation of the NLRP3 inflammasome leads to the release of downstream cytokine IL-1*β*, which blocks the reception of normal insulin signals, leading to IR [57]. In vitro, IL-1*β* facilitates lipid accumulation and cell injury in hepatocytes [58]. In the present study, HJF significantly reduced activation of the NLRP3 inflammasome and abdominal aorta serum IL-1*β*/18 levels in HFD-fed rats. Therefore, we speculate that the regulation of NLRP3 inflammasome may be one potential mechanism for HJF treatment of NAFLD.

The TCM theoretical system encompasses the concepts of holism and treatment based on syndrome differentiation, and different combinations of herbal medicines are used for different clinical characteristics and pathological manifestations. Chinese medicinal formulae, such as HJF, are composed of complex Chinese herbs under the guidance of TCM theory and applied to exert pleiotropic effects on diseases. As the etiology and pathogenesis of NAFLD are complicated and not completely clear, Chinese medicine may be an effective alternative treatment. In the present study, we observed that HJF exerted pleiotropic effects on the gut microbiota, intestinal barrier, and inflammasome for the treatment of NAFLD.

In conclusion, we demonstrated that the TCM formula HJF reduces hepatic steatosis, and this effect could be by decreasing certain gut bacteria (such as Enterobacteriaceae bacteria and* F. rappini*), alleviating intestinal endotoxemia and reducing activation of the NLRP3 inflammasome.

## Figures and Tables

**Table 1:** The composition of compound probiotics.

Ingredients	Every 100 g
*Lactobacillus casei Zhang*	≥10 × 10^10^ CFU
*Lactobacillus plantarum HM-P8*	≥10 × 10^10^ CFU
*Lactobacillus paracasei HM-P9*	≥5 × 10^10^ CFU
*Lactobacillus rhamnosus HM-R1 *	≥5 × 10^10^ CFU
*Lactobacillus acidophilus HM-A2*	≥5 × 10^10^ CFU
*Lactobacillus bulgaricus HM-B1*	≥5 × 10^10^ CFU
*Bifidobacterium lactis HM-V9*	≥10 × 10^10^ CFU
*Bifidobacterium adolescentis HM-A1*	≥5 × 10^10^ CFU
*Bifidobacterium longum HM-L4*	≥5 × 10^10^ CFU
Galactooligosaccharide	≥15 g

**Table 2:** Thecomposition of HJF.

Chinese name	English name	Part used	Proportion
Huangqi	Astragali Radix	Root	15
Hongqu	Red Rice	Rice fermented with the mould *Monascus purpureus*	12
Yinchen	Artemisiae Scopariae Herba	Aerial Part	10
Gouqizi	Lycii Fructus	Fruit	10
Jianghuang	Curcumae Longae Rhizoma	Rhizome	6
Heye	Nelumbinis Folium	Leaf	10
Houpu	Magnoliae Officinalis Cortex	Bark	6
